# Angle-resolved photoemission spectroscopy for the study of two-dimensional materials

**DOI:** 10.1186/s40580-017-0100-7

**Published:** 2017-03-29

**Authors:** Sung-Kwan Mo

**Affiliations:** Advanced Light Source, Lawrence Berkeley National Laboratory, Berkeley, CA 94720 USA

**Keywords:** Two-dimensional materials, Photoemission, ARPES, Transition metal dichalcogenides

## Abstract

Quantum systems in confined geometries allow novel physical properties that cannot easily be attained in their bulk form. These properties are governed by the changes in the band structure and the lattice symmetry, and most pronounced in their single layer limit. Angle-resolved photoemission spectroscopy (ARPES) is a direct tool to investigate the underlying changes of band structure to provide essential information for understanding and controlling such properties. In this review, recent progresses in ARPES as a tool to study two-dimensional atomic crystals have been presented. ARPES results from few-layer and bulk crystals of material class often referred as “beyond graphene” are discussed along with the relevant developments in the instrumentation.

## Introduction

Since the successful isolation of graphene [1], the efforts to understand and utilize the extraordinary properties of two-dimensional (2D) materials have been one of the central themes of condensed matter physics and materials science research. 2D materials often possess physical and chemical properties that are not attainable in their bulk counterpart. Prime examples are the massless, chiral, Dirac fermions in graphene [2, 3] and consequential high mobility [1, 4] and distinctive quantum Hall effect [4, 5].

It has been known early on that other layered materials can also be mechanically exfoliated [6] using similar techniques used for graphene. However, it was until the discovery of much enhanced photoluminescence quantum efficiency in monolayer MoS$_2$ [7, 8], far beyond those of bulk and bilayer, that the boom of research on the 2D materials “beyond graphene” was ignited. While preserving graphene’s flexibility and tunability, atomically thin layers of such 2D materials provide access to more diverse electronic and optical properties [9, 10], opening a route to functional devices with tailored properties either by themselves with suitable external perturbations [11] or through heterostructures with other 2D materials [12].

Angle-resolved photoemission spectroscopy (ARPES) provides crucial information regarding the physics behind unique electronic and optical responses of 2D materials by providing a direct picture of their momentum-resolved electronic structure. ARPES has been proven to be an essential tool to reveal in-depth information on the electronic properties of 2D atomic layers and their interfaces, such as enhanced superconductivity in FeSe on SrTiO$_3$ [13, 14], thickness dependent topological properties of Bi$_2$Se$_3$ [15], induced superconductivity by proximity effect in a topological insulator [16], and 2D electron liquid in oxide interfaces [17]. At the same time, developments of new experimental techniques expanding the boundary of ARPES provide new opportunities to explore previously inaccessible spatial, spin, and time domains. This marks the ARPES studies of 2D materials still being in an early stage with far-reaching future ahead.

This review will focus mainly on the investigation of the electronic structure of 2D materials beyond graphene using ARPES. The discussion on the synthesis, various methods of characterization, and device applications are left to be found in prior reviews [11, 18–28]. Graphene is still the most studied 2D material to this date and ARPES has played a key role in understanding its electronic properties. Since there have been many insightful reviews [29–32], the ARPES studies on graphene will not be covered in this review. Many materials discussed in this review also harbor non-trivial topological properties, such as topological insulator or quantum spin Hall insulator, and ARPES has been essential in providing experimental evidences of their topological nature. Such topological properties will be discussed as necessary but a more comprehensive reviews are available elsewhere [33–35].

In the following, a short description of ARPES technique will first be provided with a focus on the recent developments relevant to the studies of 2D materials. Reviews on each material class, various transition metal dichalcogenides (TMDCs) and elemental monolayers in group IV and V, will follow.

## Angle-resolved photoemission

Photoemission spectroscopy (PES) utilizes the photoelectric effect originally observed by Hertz [36] and successfully explained by Einstein [37]. Electrons are excited to the energy levels above the vacuum energy level by incident photons, provided the photon energy is large enough to overcome the work function. By analyzing the energy and momentum of the out-going electrons, one can reconstruct the information of their energy and momentum inside the solid or gas system [38].

**Fig. 1:**
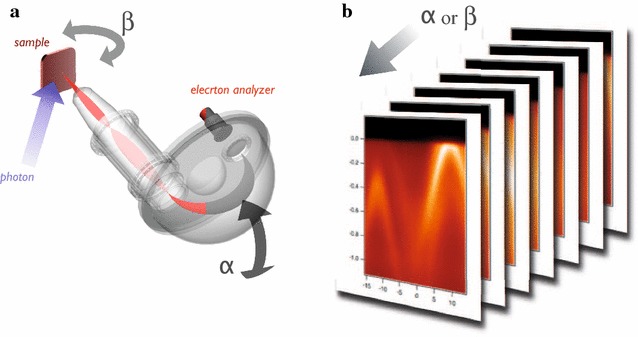
**a** A schematic of a typical setup of ARPES measurements. Photons are provided by the sources such as synchrotron light sources, discharge lamps, and laser sources. The energy and one of the in-plane momentum of out-going electrons are measured by hemispherical electron analyzer. By rotating either electron analyzer ($\alpha$) or sample ($\beta$), the momentum information perpendicular to that from the analyzer is also collected. **b** A typical set of ARPES data. A modern multichannel electron analyzer can acquire spectra in two-dimensional energy-momentum space. By combining multiple images perpendicular to the direction defined by the analyzer slit, one may construct a complete energy-momentum dispersion for in-plane momentum directions. This cube of data then can be dissected to obtain band structures along any momentum direction or iso-energy surface such as Fermi surface

The technique has been traditionally used for the chemical analysis of atoms, molecules, and solid state samples [39]. With the advent of modern synchrotron light sources and the multichannel detectors with high energy and momentum resolutions, ARPES became a standard tool to study the electronic structures of complex material systems, such as high temperature superconductors [40–42], transition metal oxides [43], graphene [30], and heavy fermions [44] (Fig. 1).

In the simplest sense, ARPES provides the momentum resolved band structures of materials in a most direct way. From an ideal system composed of free electrons in a perfect lattice, one may expect to obtain a parabolic band structure from an ARPES measurement with effective mass coinciding with the bare mass of electrons. The spectra from such measurements should produce sharp peaks in both energy and momentum directions with linewidths only limited by the experimental resolutions from the photon source and the detector. However, in the real material systems, electrons interact with other electrons, scatter from collective modes such as phonons, and often form an ordered or protected states. Such complexity leaves footprints in the ARPES spectra, such as distinct lineshapes [45, 46], modified energy-momentum dispersion or kink [47–49], deviation from the parabolic dispersion [33, 50], and opening of a gap in the energy spectrum [41, 51]. By analyzing lineshapes, dispersions, and energy gaps in detail, one may obtain information going beyond the simple electronic dispersion relation, i.e., complex scattering processes that electrons experience inside the material systems.

From the experimental point of view, the most pronounced character of ARPES is its surface sensitivity. Due to the limited inelastic mean free path of electrons [52], ARPES spectra are dominated by the signals from the first few layers of the solid with a typical choice of photon energy. A natural consequence of such surface sensitivity is that all the experiments have to be done in a ultra-high vacuum (UHV) environment $\sim$10$^{-11}$ Torr and on a clean surface either cleaved, grown, or treated freshly. This implies that ARPES is most suitable for the studies of 2D or quasi-2D layered materials, albeit it is also possible to probe three-dimensional (3D) electronic structure by varying incident photon energies with sufficient range and spacing [53, 54]. The surface sensitivity of the ARPES actually makes it an ideal tool for the study of 2D materials. For many spectroscopic and microscopic tools, the measurements of atomically thin 2D crystals are technically challenging due to an insufficient cross-section of thin layers.

Conventional ARPES systems developed in the last 20 years or so, however, is not completely well-suited for the studies of graphene-like 2D materials. The most limiting factor is the size of the materials. A typical size of exfoliated 2D crystal flakes are roughly 1–10 $\upmu$m, which is much smaller than the typical beam spot size from synchrotron light source $\sim$25–100 $\upmu$m. One also needs to search for a flakes with a desired layer number (done by optical microscopy for, e.g., transport measurements) and shine the light for ARPES measurements exactly on the same spot, which is not trivial under UHV environment. Either by enlarging the sample by growing them using epitaxial methods, or by collimating the photon beam to the size comparable to the samples, the ARPES becomes accessible for the studies of 2D materials. It is not surprising only after successful growth of graphene from SiC [55, 56], graphene has become available for ARPES and other spectroscopic tools.

In the next few paragraphs, efforts to bring 2D materials to ARPES will be described along with a few other recent developments in ARPES technique relevant to the 2D material research.

*MBE+ARPES* The 2D materials described in this review can be synthesized with various techniques other than mechanical exfoliation, such as chemical vapor deposition (CVD), physical vapor deposition (PVD), liquid phase deposition and exfoliation, and molecular beam epitaxy (MBE) [57]. MBE has advantages over other methods for the ARPES measurements, since it can produce large area samples with well-aligned domains without involving water or chemicals that are not UHV compatible. It also allows a layer-by-layer control of the film thickness when the growth conditions are well-refined and the process is carefully monitored with reflection high-energy electron diffraction (RHEED) [58, 59]. When the bonding between layers are mainly from weak van der Waals force, which is the case for many 2D materials such as TMDCs, many constraining conditions for MBE growth—symmetry matching and lattice matching between the substrate and the film—are relaxed, which results in a favorable condition for the MBE growth [21]. Both MBE and ARPES requires UHV systems for proper operations. Therefore it is natural to combine MBE and ARPES systems with well-designed sample holders and sample transfer schemes. There are quite a few commercially available systems as well as custom-built systems in operation in both synchrotron and laboratory environment.

**Fig. 2:**
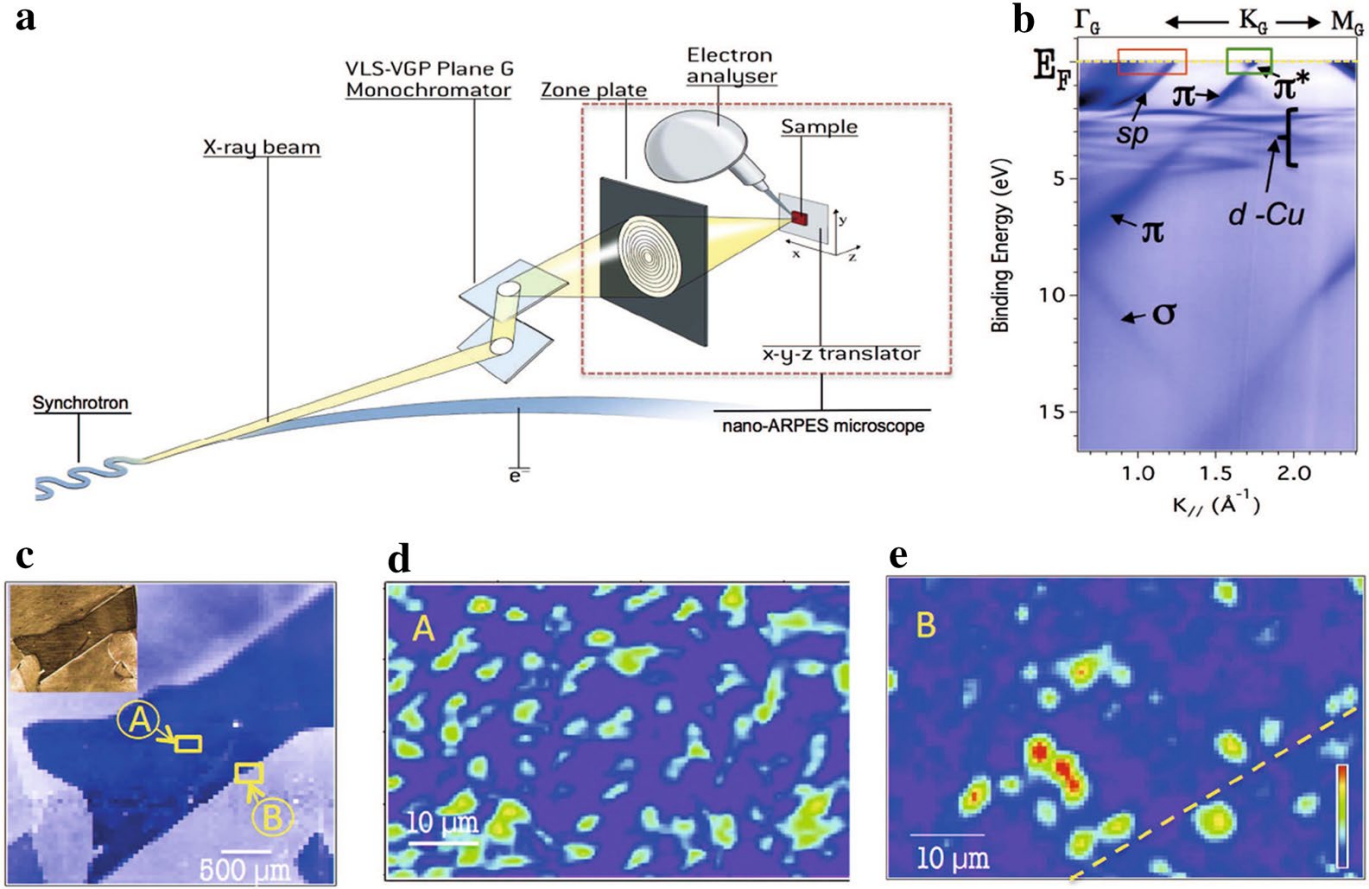
**a** A schematic of the zone plate based nanoARPES system. **b** Micro-ARPES data from graphene on copper substrate. **c** Real space image of copper state obtained by nano-ARPES mapping. Inset is an optical image of the same sample. **d**, **e** Real space image of graphene grains by measuring graphene state at the* A* and* B* positions indicated as *yellow boxes* in **c**, respectively. The figure is reproduced from Ref. [65] with permission

*nano-ARPES* The need to reduce the beam spot size for ARPES measurement is not limited to the study of micrometer sized, exfoliated 2D crystals. Complex materials often inherently possess inhomogeneity, such as domains, boundaries, and interfaces, which could play a crucial role realizing their novel properties [60]. Measuring electronic structures at multiple length scales, therefore, is an important step to fully understand the electronic properties of materials. The sub-micrometer collimation of extreme ultra-violet (XUV) and soft X-ray light is a challenge for conventional Kirkpatrick-Baez (KB) optics. Currently, there are two main schemes to achieve nano-ARPES—Schwarzschild optics and Fresnel zone plate (FZP). Spectromicroscopy beamline in Elettra [61, 62] uses multilayer coated Schwarzschild optics, which gives a large working distance from the focusing optics to the sample, compared to FZP. However, the choice of photon energy is defined by the optics geometry and limited to a few selected ones. More recently developed beamlines for nano-ARPES in ALS (MAESTRO), Soleil (ANTARES), and Diamond (I05) synchrotrons utilize the FZP (Fig. 2) to achieve 50–200 nm beam spot size [63, 64]. Most of the early results from these beamlines have been on the inhomogeneous electronic structures of 2D materials, such as graphene (Fig. 2) [65–68].

*spin-ARPES* Many 2D materials naturally break inversion symmetry when the thickness is reduced to a few layers, thus harbor a platform to host a large Rashba type spin splitting when the spin-orbit coupling (SOC) is strong enough [69]. In addition, quite a few of them possess non-trivial topological index, which results in well-defined spin textures in their band structures [70]. Spin-resolved ARPES (spin-ARPES) is a probe to directly show the spin texture of a particular band [71, 72]. The caveat here is that the delicate spin-ARPES matrix element, dependent on the photon energy, polarization, detection geometry, and so on, should be considered in great detail for a proper interpretation of measured spin dependent ARPES signal [73, 74]. A standard experimental setup engages multiple pairs of Mott detectors, which utilizes the spin-orbit interaction in the process of electron scattering off heavy elements targets (Au, Th, etc.) [72]. This process is inherently inefficient compared to the regular ARPES by a few orders of magnitude, thus makes spin-ARPES a photon-hungry experiment. Recent developments of multi-channel spin detectors using various schemes [75–78], with much improved detection efficiency, promise a better understanding of non-trivial spin textures from materials as well as the spin-ARPES process itself.

*tr-ARPES* Time-resolved ARPES (tr-ARPES) is a hybrid of pump-probe optical spectroscopy and ARPES. By applying ultrafast laser pulses to perturb the equilibrium state, the subsequent ARPES measurements can probe the dynamics of transient state or the unoccupied states of the band structure [79, 80]. A typical setup involves a pump laser $\sim$1.5 eV and a probe beam $\sim$6–7 eV, the latter being driven by a high-harmonic-generation (HHG) source. The 6–7 eV photon source has been limiting the field of view in *k*-space only around the $\varGamma$ point of the typical Brilloiun zone (BZ). Recent developments in HHG reaching XUV range with high flux and high repetition rate [81–83] could bring a revelation in studying the electronic structure of 2D materials, since it allows one to reach, e.g., the *K* point of hexagonal BZ of TMDCs. There also has been the effort to combine both time and spin resolved ARPES and some of the early results start to appear in literature [84].

## ARPES studies on TMDC

### 2*H*-MoS$_2$, WS$_2$, MoSe$_2$, WSe$_2$

The semiconducting 2*H*-phase TMDCs MX$_2$ (M = Mo, W; X = S, Se) are arguably the most studied 2D materials beyond graphene. They have attracted intense interest since the observation of much enhanced photoluminescence in monolayer MoS$_2$ [7, 8], which is interpreted in terms of a transition from an indirect gap semiconductor to a direct band gap semiconductor when the thickness is reduced to the monolayer. The interesting physical property is not limited to the band gap transition and potential applications based on that, such as photoharvesting [85, 86] and photodetection [87] devices. It includes the spin-splitting of the valence band (VB) [88, 89], a well-pronounced valley degree of freedom [90–93], a charge density wave (CDW) state at the mirror twin domain boundaries [94], as well as the gigantic exciton binding energy that governs their optical spectra [95–97].

**Fig. 3:**
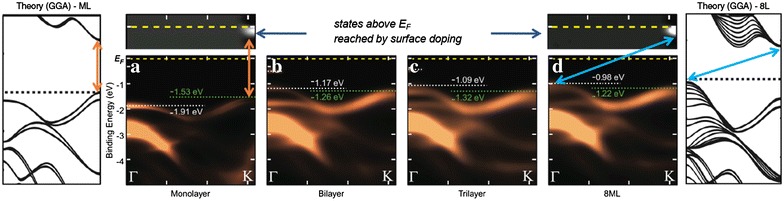
ARPES data of MBE grown MoSe$_2$ with **a** monolayer, **b** bilayer, **c** trilayer, and **d** 8-layer thicknesses. The *left* and *right most panels* show the theory calculations for monolayer and 8-layer, respectively. *Orange* and *light blue arrows* indicate direct band gap from *K* to *K* point and indirect band gap from $\varGamma$ to *K* point, respectively.* Two top panels* above **a** and **d** are the conduction band minimums for monolayer and 8-layer, respectively, which is brought down in energy by the chemical potential shift through surface potassium doping. One need to note, however, the surface doping may also modify the band structure. The figure is rearranged from the same data set used for Ref. [98]

ARPES can directly show the changes in electronic structure when the thickness is varied from monolayer to thicker layers. Figure 3 summarize the results from Ref. [98], in which the layer-by-layer band structure evolution is directly mapped using *in situ* ARPES on MBE grown MoSe$_2$ samples on bilayer graphene substrate. The main feature of the band structure variation is the change in the number of the $\varGamma$ point branches and concomitant change of the VB maximum from *K* to $\varGamma$ when thickness increases from monolayer to bilayer. With the conduction band being at the *K* point regardless of the number of layers, as probed by chemical potential shift through surface potassium doping, this immediately indicates a transition from a direct band gap semiconductor to an indirect band gap semiconductor. These observations match well with the first principle calculations [88, 98], although exact position of the conduction band minimum varies depending on the calculation methods [99]. Another important aspect is the spin splitting at the *K* point ~180 meV for MoSe$_2$ and $\sim$470 meV for WSe$_2$ [100]. The latter is larger due to the stronger SOC induced by a heavier element W. ARPES studies on epitaxial grown 2*H*-MX$_2$ films can be found for WSe$_2$ on bilayer graphene [100], MoS$_2$ on Au(111) [101, 102], and WS$_2$ on Au(111) [103]. They also report the effect of surface potassium doping, which is not only shifts the chemical potential but modifies the band structure [104, 105] allowing yet another parameter to control the electronic structure of semiconducting TMDCs.

There have been quite a few ARPES studies on the bulk samples of 2*H*-MX$_2$ samples [104, 106–111]. The issue here is to determine the band parameters precisely, such as the size of the electron removal gaps at the $\varGamma$ and *K* points and the size of the spin-splitting at the *K* point, and to figure out the origin of apparent discrepancies among the values from different probes and different theory calculations. One notable finding is that the size of the band gap and the position of the conduction band minimum is extremely sensitive to the *c* / *a* ratio [99, 110] of the lattice constants.

Mechanically exfoliated samples and CVD grown samples of 2*H*-MX$_2$ and their heterostructures with other 2D materials have been successfully measured with nano-ARPES [99, 106, 112–118]. While the spectra from nano-ARPES do show the essential features expected from the band structure of few-layer 2*H*-MX$_2$, they also suffer from the limited resolution by allowing maximum photon flux to compensate the loss caused by the focusing optics. On the other hand, nano-ARPES gives a much more flexibility in studying the heterostructures of 2D materials, expanding the boundary of sample preparation for ARPES measurements. Of particular interest, nano-ARPES studies on the graphene/MoS$_2$ heterostructure [114, 115] report a strong hybridization between MoS$_2$ and graphene, which is evidenced from the opening of hybridization gaps in the bands located at high binding energies. This is not consistent with the ARPES data on MBE grown MoSe$_2$ on graphene [98], for which such hybridization has not been found, suggesting that the interaction between 2D materials in MBE-grown and mechanically-produced heterostructures may not be exactly the same. The work in Ref. [118] focuses the band alignment between MoSe$_2$ and WSe$_2$ bands in a monolayer MoSe$_2$/monolayer WSe$_2$ heterostructure, which provides essential information for the understanding of the charge transfer process in a device made with such heterostructures [119].

**Fig. 4:**
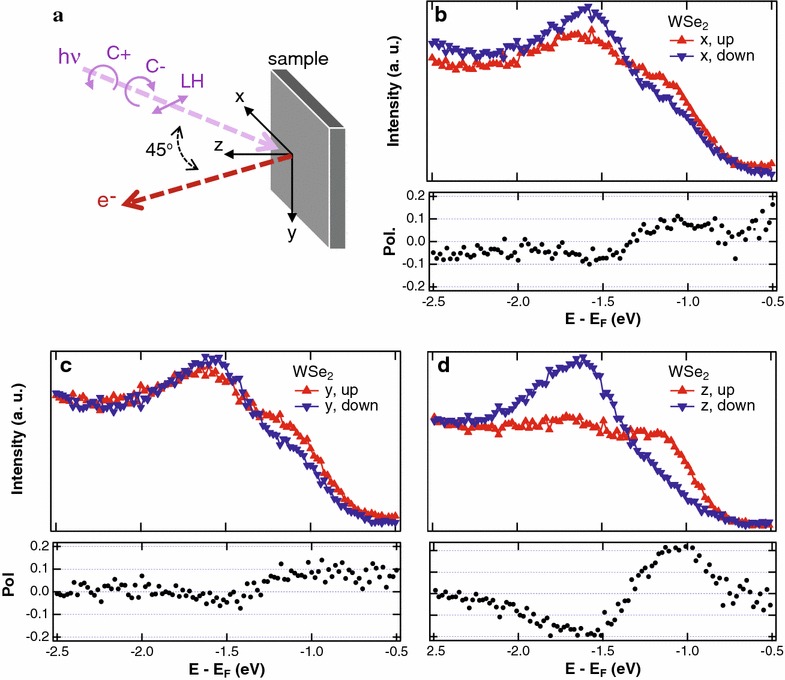
Spin-ARPES data of MBE grown WSe$_2$ at the *K* point of hexagonal BZ. **a** A schematic of measurement geometry. **b**–**d** Spin resolved energy distribution curves at *K* for **b** *x*, **c** *y*, **d** *z* directions defined in **a**. The figure is reproduced from Ref. [123] with permission

A distinct valley degrees of freedom with completely flipped spin textures between *K* and $K'$ points of the hexagonal BZ is the basis of the valleytronic applications in MX$_2$. Spin-ARPES must provide a direct view of the spin texture and the spin flip between different valleys. Earlier spin-ARPES works are essentially measured on bulk samples of MX$_2$, such as bulk inversion symmetry broken 3R phase of MoS$_2$ [120] and spin polarization induced by the space charge residing in the overlayer [121]. Riley et al. [122] report spin polarized signal just from a regular bulk WS$_2$, and interprets their results in terms of strong electron localization at the *K*/$K'$-point of WS$_2$. Spin-ARPES data on MBE grown MoSe$_2$ and WSe$_2$ [123] find a mostly out-of-plane spin polarization and confirm that the splitting at the *K* point is indeed a spin-splitting (Fig. 4). However, the spin-flip between *K* and $K'$ was not clearly exhibited due to the intricate interplay among spin, orbital, and spin-ARPES matrix elements.

There are only handful of tr-ARPES data on 2*H*-MX$_2$ systems since the development of XUV probe is still in its early stage. The tr-ARPES data on the bulk 2*H*-MoS$_2$ [124] found multiple decay channels of optically populated electrons in the conduction band with largely different time scales $\sim$1 to $\sim$15 ps. The tr-ARPES work on the epitaxially grown MoS$_2$ on Au(111) [125] and on graphene [126] report a renormalized band gap size depending on the interaction of electrons with the substrate as well as much faster electron dynamics compared to that in bulk.

### $1T'$-MoTe$_2$, WTe$_2$, MoSe$_2$, WSe$_2$

$1T'$ phase TMDCs in their bulk form has been studied intensely in recent years since the discovery of non-saturating linear magnetoresistance (MR) in WTe$_2$ [127] and the prediction of the type-II Weyl semimetals [128, 129]. There have been a surge of research following these discoveries including ARPES data from multiple groups, either focusing on the large anomalous MR [130–133] or their topological properties [134–142]. The competing pictures to explain anomalous MR are electron-hole carrier compensation [143] and topological protection [144, 145]. The latter is closely related to the topological classification of $1T'$ TMDCs. ARPES measurements should in principle provide an unambiguous answer to these issues by deciding the sizes of electron and hole Fermi surfaces (FS) and revealing the surface Fermi arcs. However, a rather closely packed multiple Fermi pockets in a narrow momentum region near $\varGamma$ [131] and the Fermi arc being slightly above $E_\mathrm{F}$ [128] hinder a clear observation of various pockets and arcs. Furthermore, it has been noted that the classification of arc-like features and assignment of Weyl nodes and their projection on the surface are closely dependent on the theoretical models, which in turn is very sensitive to the parameters such as lattice constants [136].

The 2D limit of $1T'$ phase of TMDCs hold interesting theoretical proposal that it may host a large gap quantum spin Hall (QSH) insulator phase [146], suggesting a thickness dependent changes in the topological nature of these materials. A band gap opening, with a size similar to the theoretical prediction, in the optical and transport measurements of MoTe$_2$ alluded that such proposal may be true [147]. Recent transport measurements suggesting an edge conduction below critical temperature in WTe$_2$ may further provide evidences of the existence of QSH insulator phase in monolayer $1T'$ TMDCs [148]. Up to now, there has not been any ARPES data on the QSH aspects of monolayer $1T'$ phase TMDCs. A direct observation of the band gap opening at the low temperature, the orbital characters of inverted bands, and the momentum resolved electronic structure would benefit a development of a comprehensive picture for this intriguing material class.

### NbSe$_2$, TiSe$_2$

TMDCs with transition metal elements such as Nb, Ti, Ta, and V share a common theme of physics stemming from the coexisting collective orders of charge density wave (CDW) and superconductivity (SC) [149]. Whether these orders compete or cooperate, particularly at a few layer limit, is an interesting question with significant implications, for example, to the physics of high temperature superconductivity in cuprates [150].

**Fig. 5:**
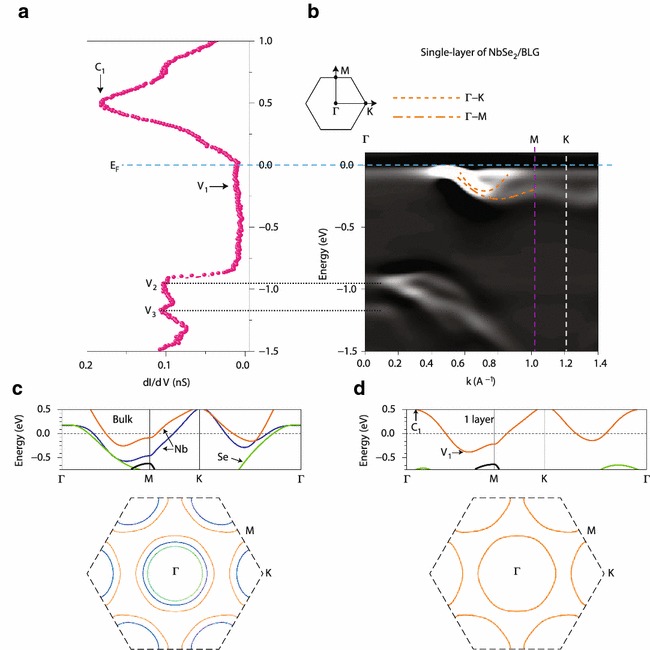
Electronic structures of NbSe$_2$ on bilayer graphene from **a** STS, **b** ARPES, and first principle calculations for **c** bulk and **d** monolayer. MBE grown samples possess two distinct domains with 30$^{\circ }$ angle between each other to overlap the dispersions along $\varGamma$-*K* and $\varGamma$-*M* directions in a single ARPES spectrum. The figure is reproduced from Ref. [155] with permission

2*H*-NbSe$_2$ is a prototypical system to study the coexisting CDW and SC orders with $T_\mathrm{CDW}$ $\,=\,33$ K and $T_\mathrm{C}$ $=$ 7.2 K in its bulk form. There have been multiple ARPES studies on the bulk NbSe$_2$ [151–154], focusing mostly on the mechanism of the CDW order formation. A combined ARPES and scanning tunneling microscopy/spectroscopy (STM/STS) study on a MBE grown monolayer NbSe$_2$ on bilayer graphene reveals that the band structure fundamentally changes when the thickness is reduced to the monolayer going from a multi-band FS to a single-band FS (Fig. 5) [155]. While the band structure changes, the 3 $\times$ 3 CDW order remains intact in both ordering *q*-vector and the transition temperature. The SC order is still present but the $T_\mathrm{C}$ is suppressed down to 1.9 K. This makes a contrasting case with the Raman study [156] on exfoliated NbSe$_2$, for which a large increase of the CDW transition temperature was observed with a similar suppression of $T_\mathrm{C}$. It is also in discord with the interpretation of transport data under magnetic field [157] and the spin-ARPES measurement on bulk [158], both of which assumes the spin-momentum locking based on the preservation of the same band structure around *K*-point even in the monolayer limit. A possible origin of such discrepancy could be the role of substrate, which should be demonstrated by further studies. Recent MBE $+$ ARPES measurements on NbSe$_2$ report that it is possible to isolate either 2*H* or 1*T* phase depending the substrate temperature during the growth process [159].

1*T*-TiSe$_2$ makes a 2 $\times$ 2 $\times$ 2 CDW order below $T_\mathrm{CDW}$ $\approx 200$ K and the superconductivity can be induced by suppressing CDW order through, e.g., electric field, doping or pressure [160–162]. Despite multiple ARPES studies and theoretical scenarios to explain the nature of the bulk CDW phase [161–166], the microscopic origin still remains elusive. ARPES studies on few-layer samples should provide a systematic evolution of the CDW by suppressing and reinstating the order along the *c* axis. Chen et al. report [167, 168] an enhanced CDW transition temperature $T_\mathrm{CDW}$ $=$ 232 K from the monolayer TiSe$_2$ grown on bilayer graphene with 2 $\times$ 2 superstructure. The layer-by-layer ARPES investigation also find that there exist two distinct transition temperatures in the bilayer, one which is similar to that of monolayer and the other similar to that of bulk. These two transition temperatures conform to the bulk value $\approx$200 K for the films thicker than 3 layers. Their findings are in contrast to the similar study by Sugawara et al.  [169], in which an essentially the same $T_\mathrm{CDW}$ has been reported in the monolayer TiSe$_2$ on graphene. It is worthwhile to note that the bulk 1*T*-TiSe$_2$ and TiS$_2$ have been the model system to study the dynamics of CDW order using tr-ARPES [170, 171]. The time scale for the melting of the CDW order was found to be extremely small $\sim$20 fs, which has been attributed to the screening by the transient generation of free charge carriers [170].

## ARPES studies on group IV 2D materials

### Silicene

Silicene is a graphene analog of silicon in a low-buckled honeycomb lattice [172, 173]. Since the theoretical studies that predict [174, 175] the existence of a stable silicene phase with a massless Dirac fermion and a Dirac point at $E_\mathrm{F}$, it received a large amount of interest. The freestanding silicene has not been isolated experimentally, yet a few layer silicon films on various substrates, such as Ag(110) [176], Ag(111) [177–181], ZrB$_2$ [182, 183], Ir(111) [184], and MoS$_2$ [185], have been synthesized. Care should be taken in the interpretation of the ARPES data since the silicon layer itself forms various reconstructed phases depending on the growth conditions and the interaction with the substrate could affect the electronic structure.

The most studied silicene structure is monolayer Si on Ag(111) surface. A combined ARPES and STM study by Vogt et al.  [177] reported a Dirac cone like linear band structure from a (4 $\times$ 4) Si overlayer on Ag(111). The estimated $v_\mathrm{F}$ is similar to that of graphene, although there exists an electron removal gap opening $\sim$0.3 eV resulting from the interaction with the substrate. Such interpretation was later challenged by ARPES studies [180, 181] that showed a saddle point like dispersion rather than a conical one at the *K* point of Si monolayer at which the Dirac cone was reported in the previous studies. Conflicting ARPES data were also reported for Si multilayer on Ag(111). De Padova et al. [186] reported a growth of Si multilayers with ($\sqrt{3} \times \sqrt{3}$)R$30^{\circ }$ reconstruction and ARPES data that shows linear dispersion with a Dirac point at 0.25 eV binding energy. Mahatha et al. [187] argued that such band should be attributed to the Si-modified Ag(111) interface state and the Si films of a few monolayer thickness on Ag(111) are $sp^3$ diamondlike Si rather than multilayer silicene. Similar opening of the gap and deviation from the Dirac dispersion due to the buckling and a substrate interaction have been observed for silicene on ZrB$_2$ [182, 183].

### Germanene

Germanene is a sister material of silicene with a similar low-buckled honeycomb lattice structure [173, 175]. Compared to silicene, the study on germanene is in its early stage due to the sample availability. Synthesis of germanene on metal substrates have only been reported recently—on Pt(111) [188], Al(111) [189, 190], Ag(111) [191], and Au(111) [192]. A report has been made on the synthesis of germanene on MoS$_2$ [193]. Most of the studies utilize STM and STS to identify structural properties with local electronic structure. Up to now, ARPES measurement has been rare. Few layer germane on Au(111) is reported to possess Dirac cone like linear dispersions in ARPES spectra [192].

### Stanene

Out of group IV elemental 2D materials, stanene, a single layer of tin, is expected to possess the largest SOC. This implies, with the buckling structure of honeycomb lattice, a more textured topological properties are possible, including the prediction of a large gap QSH insulator [194, 195] with a gap size larger than 100 meV for room temperature applications [196].

Few layer $\alpha$-Sn films have been grown on InSb substrate and investigated using ARPES [197, 198]. Both results confirm the existence of topological surface state with a linear dispersion of Dirac cone. They also directly observe the spin polarization in the topological surface state through spin-ARPES measurements. Barfuss et al. find that the role of strain is essential to realize the topological insulator phase by density functional and GW calculations. Ohtsubo et al. report that the topological character in $\alpha$-Sn depends on thickness and it becomes a topological insulator only within a small range of thickness.

**Fig. 6:**
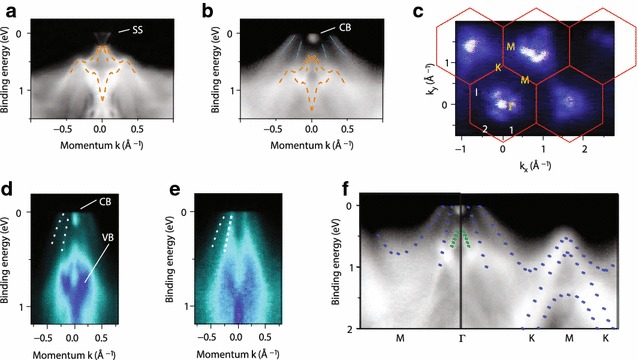
Electronic structures of stanene on Bi$_2$Te$_3$. **a**, **b** ARPES spectra of bare Bi$_2$Te$_3$(111) and stanene on Bi$_2$Te$_3$ along *K*-$\varGamma$-*K* direction, respectively. The *orange dashed lines* indicate the bulk bands of Bi$_2$Te$_3$ substrate. **c** FS intensity map. The *red hexagons* are the 2D BZ of stanene. **d**, **e** ARPES spectra around $\varGamma$ taken with *p*-polarization and *s*-polarization, respectively. *White dotted lines* denote the hole bands of stanene. CB and VB mark the conduction and valence bands of Bi$_2$Te$_3$. **f** ARPES spectra along $\varGamma$-*M*-$\varGamma$-*K*-*M*-*K* directions. *Blue dotted lines* indicate the experimental band structure of stanene and the *green dashed lines* are one of the hole band of Bi$_2$Te$_3$. The figure is reproduced from Ref. [199] with permission

Stanene, a 2D structure of $\alpha$-Sn(111), has only recently been realized by MBE on Bi$_2$Te$_3$(111) substrate [199]. The crystal structure has been identified in STM as that of stanene. The electronic structure was investigated by ARPES (Fig. 6). The measured band structure bears a close resemblance with the DFT calculation that includes a contribution from Bi$_2$Te$_3$ substrate. However, the measured band structure shows a complete disappearance of $p_z$ band near the *K*-point, which indicate that $p_z$ orbitals are fully saturated by an interaction with the substrate or an inclusion of adsorbates [195, 199]. At the same time, the chemical potential is shifted to bring the hole bands around the $\varGamma$-point to cross $E_\mathrm{F}$. This makes the identification of the predicted QSH phase [194, 195], a band inversion and an opening of a gap, impossible for ARPES.

## ARPES studies on group V 2D materials

### Phosphorene

Phosphorene is a 2D atomic crystal made of black phosphorus [200]. It can be exfoliated from the bulk crystal and retains the direct band gap ranging 0.3–1.7 eV. The size of the band gap fills the gap between those of graphene and semiconducting TMDCs. The gap size can be controlled through the number of layers, strain, and electric field [200–202]. The atomic structure of black phosphorus is highly puckered, armchair shape in one direction and zigzag shape in the perpendicular direction. This results in an unusual mechanical properties and makes phosphorene a ideal template for an atomic chain [200].

**Fig. 7:**
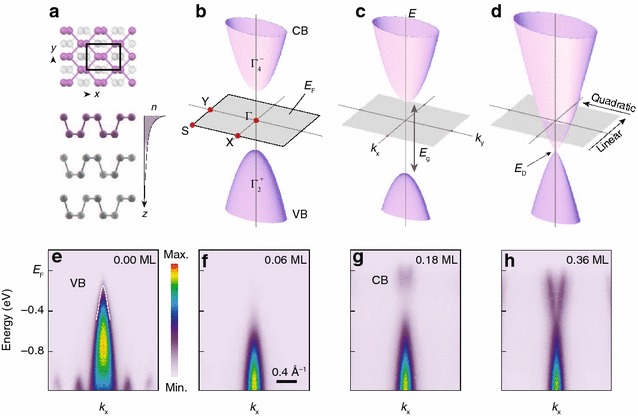
**a** Atomic structure of black phosphorus. **b**–**d** A schematic of electronic structure evolution in black phosphorus with surface electron doping. **e**–**h** ARPES data along $\varGamma$-*X* direction as the chemical potential and the band structure change with surface electron doping. The figure is reproduced from Ref. [205] with permission

There have been a number of ARPES studies [202–206] on bulk black phosphorus. Han et al. [204] made a wide range photon energy dependence to decide the $k_z$ dispersion and find that the valence band maximum occurs at the *Z*-point of BZ. They also found that experimental electronic structure from ARPES is more two-dimensional compared to the theoretical calculations. Kim et al. [205] made interesting observations when potassium is dosed on the surface of black phosphorus (Fig. 7). With the increasing amount of potassium on the surface, the band structure of black phosphorus can be tuned from a direct band gap semiconductor with variable gap size into a band-inverted semimetal. At the critical point of such transition, the electronic state becomes highly anisotropic, with a linear Dirac dispersion along the armchair direction and nearly parabolic dispersion along the zigzag direction. Theoretical studies predict [207] that further surface doping on few-layer black phosphorous would turn the system into a Dirac semimetal with linear dispersion in all three momentum directions.

Until now, the investigation of electronic structure on few-layer black phosphorus has been lacking on the ARPES side. The main bottleneck has been the difficulty in sample preparation due to the fast degradation of the material [200]. Encapsulation of exfoliated samples, a technique readily available to, for example, optical measurements [208], is not compatible with ARPES due to the short escape depth of the photoelectrons.

### Arsenene, antimonene, and bismuthene

Down the row in the periodic table from phosphorus, arsenene, antimonene, and bismuthene are the 2D forms of arsenic (As), antimony (Sb), and bismuth (Bi), respectively. These materials are expected to assume a stable form of atomic structure out of multiple allotropes of their bulk counterpart and to stay stable in room temperature and ambient environment [209, 210]. Shared themes of material properties of these 2D crystals are the semimetal to semiconductor transition with reduced dimension with variable gap size through strain and other parameters [209, 210] and the diverse topological phases that is particularly pronounced for antimonene and bismuthene [210, 211]. While the isolation of arsenene is yet to be realized, antimonene and bismuthene has been successfully exfoliated [212, 213] and grown by epitaxial methods [214–218].

ARPES measurements have been performed on a single layer Sb grown on Sb$_2$Te$_3$(111) and Bi$_2$Te$_3$(111) [215]. It has been shown that the band structure of Sb overlayer is strongly modified due to the substrate effect. A single layer Bi has attracted interest as a candidate material for the realization of a QSH insulator [216, 217, 219]. STM combined with ARPES measurements on Bi(111) single layer grown on Bi$_2$Te$_3$(111) [216] reported signatures of the topological edge state albeit $E_\mathrm{F}$ has been shifted deep into the valence band. STM measurements on the step edge of Bi single crystals [219] also found an edge state above $E_\mathrm{F}$. More recent study on Bi(111) single layer grown on SiC(0001) [217] with STM and ARPES indeed observed an edge state lying in a band gap as large as ~0.8 eV. A sizable tensile strain of 18% is identified as essential to realize a large band gap QSH phase, which stress the importance of substrate in realizing desired physical properties.

## Summary and outlook

The ARPES study of 2D materials beyond graphene is still in its early stage. A crucial element of connecting ever expanding 2D material classes to a UHV spectroscopic tool such as ARPES is a tight integration of sample synthesis and characterization. This has been only recently realized with embedded MBE systems in synchrotron and laboratory based ARPES systems, as well as nano-ARPES with state-of-art sample synthesis and preparation tools. In addition to the availability of samples, the spin and time domain with revolutionary detectors and laser probes would expand the range of physics that can be explored through ARPES.

On the material side, many new 2D materials with exotic properties are being theoretically proposed and experimentally realized. Growth and synthesis methods are being aggressively investigated and refined. For example, this review did not include the recent exciting developments in monochalcogenides and monopnictides, such as remarkable thermoelectricity in SnSe [220], robust ferroelectricity at room temperature in few-layer SnTe [221], high mobility and quantum Hall effect in few-layer InSe [222], large gap QSH phase in functionalized InBi monolayer [223, 224]. As the developments in theory and synthesis are closely linked to the electronic structure investigation utilizing the advances in ARPES instrumentation, interesting new physics to further enhance our understanding of 2D materials as well as new applications based on our discoveries are waiting to be explored.
